# Polyvinylidene fluoride/sulfonated graphene oxide blend membrane coated with polypyrrole/platinum electrode for ionic polymer metal composite actuator applications

**DOI:** 10.1038/s41598-019-46305-6

**Published:** 2019-07-08

**Authors:** Inamuddin, Heba Abbas Kashmery

**Affiliations:** Chemistry Department, Faculty of Science, King Abdulaziz University, Jeddah, 21589 Saudi Arabia

**Keywords:** Composites, Electronic properties and devices

## Abstract

A polyvinylidene fluoride, sulfonated graphene oxide composite membrane coated with polypyrrole (Ppy) and platinum metal (Pt) was fabricated. The Fourier-transform infrared (FTIR) spectroscopic analysis was done to analyze the functional groups present in the composite material. Deposition of PPy/Pt electrode and surface morphology of PVDF/SGO/Pt/PPy was confirmed by scanning electron microscopic (SEM) images. The capacity of ion exchange and proton conductivity (PC) of PVDF/SGO/Pt/PPy were 1.4 meq g^−1^ of dry ion exchanger and 4.251 × 10^−2^ S cm^−1^, respectively. A two-link flexible manipulator based on the fabricated ionic polymer metal composite (IPMC) membranes was also developed where the electromechanical behaviour of a polymer-based actuator provides an important step in robotics applications.

## Introduction

Electro-dynamic polymers (EAP) based ionic polymer metal composites (IPMCs) regularly known to be ‘artificial muscles’ are advanced options in biomedical frameworks and naturally inspired robots^1,2^. IPMC is an electroactive polymer (EPA) comprising of an ionomeric polymer or composite membrane plated by two metal electrodes^3,4^. A voltage given to the membrane electrodes produces an electric field that starts the activation in actuator film^5–7^. IPMC incited grasping has turned into a thriving exploration zone during the previous decade. A few new ‘pincher’ component as suggested by Deole and Lumia^8^ to control the little protests in wet and dry situations were developed^9^. With a specific end goal to set up the utilization of IPMC as a consistent actuator segment in delicate mechanical grippers, this is of fundamental significance to build up a compelling displaying procedure for accomplishing wanted power reaction and diversion. Because of vast scale deflections with humble working voltage and capacity to display biomimetic adaptability, in opposition to customary actuator like engines, IPMCs have raised the possibilities of their reception in exactness smaller scale holding^3,6,7^. In spite of its slower reaction time, a few unpredictable plans and work have been done on the outline and improvement of EAPs based smaller scale gripper and robots. The chief target of this study is to synthesize and developed polymeric composite material for the fabrication of IPMC membrane, and use it to outline effective gripper for taking care of smaller objects. Thus, IPMC actuator was developed using PVDF and sulfonated graphene oxide composite membrane coated with polypyrrole (PPy) layer through *in-situ* oxidative-polymerization followed by deposition of platinum metal electrodes on both sides of the membrane. The PVDF is a commercial ionomeric material. Due to its certain advantageous properties such as flexibility and plasticity PVDF has been studied for use in sensing^10^, energy harvesting^11^, vibration control^12^, membrane separation^13^ and many more^14^.

Graphene (GR)^15^ has an exceptional extended surface, high Young’s modulus, and excellent thermo-mechanical stability. Hence, it is widely used in the fields of catalysis^16^, sensors^17^, actuators^18^, nanoelectronics^19^, and storage and energy conversion^20^. After incorporation of graphene into the ionomeric polymer, it may alter its sp^2^-hybridized carbon skeleton leading to the conversion of whole material into a protonic conductor which may enhance the bending actuation performance of the actuator to many folds^21^. Due to continuous bending performance of IPMC, there may be a surface crack on the terminal surface which brings down the execution of IPMC film with the reiteration of times. To maintain a strategic distance from this downside, the coating of electrically conducting materials such as polypyrrole (PPy) and polyaniline over the surface of ionomeric membrane surface may upgrade the actuator execution by stopping the creation of ‘mud breaks’^22^.

For the better performance of the IPMC membrane, it is essential to have the high ion exchange capacity (IEC), proton conductivity (PC) and water uptake (WU). Therefore, a composite membrane of PVDF and SGO coated with Ppy was prepared to develop an ionomeric polymeric composite membrane for bending actuation applications. A combination of the large surface area and electrical conductivity, of the individual SGO and PPY, resulted in the good physical properties and high electrochemical actuation performance. The possessions of the previously mentioned parameters were followed using the fundamental characterization of IPMCs such as solvent uptake, ion exchange capacity and proton conductivity and electromechanical measurements and were compared with the reported IPMCs. After characterization of the developed ionic polymer composite membranes, it was used to develop a two-link flexible manipulator for robotic assembly, where PVDF/SGO/Ppy/Pt ionic polymer metal composite was used as an active joint link actuator for micromanipulation.

## Experimental

### Materials and measurements

Poly(vinylidene fluoride) (PVDF) beads or pallets, and N,N-dimethylacetamide ≥ 99% (NMA) (Sigma Aldrich, USA), tetra-amine platinum(II) chloride monohydrate [Pt(NH_3_)_4_Cl_2_.H_2_O (Crystalline)] (Alfa Aesar, USA), NH_4_OH (25%), HCl 67% and sodium nitrate (Merk Specialties Pvt Ltd., India), sodium borohydride (NaBH_4_) (Loba Pvt. Ltd. India) and all other materials were used as received without further purification. The aqueous solutions of HNO_3_ (1 M), NaNO_3_ (1 M), HCl (2 M), ferric chloride (0.1 M), tetraamine platinum (II) chloride monohydrate (0.04 M), NH_4_OH (5.0%) and NaBH_4_ (5.0%) were prepared using demineralized water (DMW). A solution of pyrrole in toluene (33.33%, v/v) was prepared.

Surface and cross-sectional morphologies of PVDF/SGO/PPy/Pt IPMC membranes were studied using a scanning electron microscope (SEM) (SEM Jeol, JSM-6510LV, Japan)^23–25^. The Fourier transform infrared spectroscopy (FTIR) spectra were recorded to investigate the functional group of SGO, PVDF/SGO/PPy/Pt and PPy on KBr pellets in the range of 500–4000 cm^−1^ using Perkin Elmer FTIR Spectrometer^26,27^. The electrical parameters of the PVDF/SGO/Pt and PVDF/SGO/PPy/Pt IPMC membrane were investigated using cyclic voltammetry curve with Autolab 302 N modular potentiostat/galvanostat. The mechanical stability was determined by a universal testing machine (Model: H50 KS, Shimadzu Corp.), with the 25 mm gauge length between the grips under the testing speed of 5 mm min^−1^. The tip displacement behaviour was analyzed by determining the stepwise bending response along with maximum tip displacement. A laser displacement sensor (Model: OADM 20S4460/S14F; Baumer Electronic, Germany) was used to measure the successive steps of bending response and deflection hysteresis behaviour at 0–4 V DC (**Direct current)**. Bending response with time at 5 V was also carried out. A deflection vs. force behaviour was also carried out, and multiple experiments were conducted for load characterization to find out the repeatability using a digital load cell (Model: Citizen CX-220, Make: India).

### Synthesis of sulfonated GO

The GO was synthesized using a modified Hummer’s method^28^.

### Preparation of PVDF/SGO/PPy/Pt IPMC membranes

Preparation of ionomeric membranes was started by dissolving 2.5 g dried PVDF beads in 50 mL N, N-dimethylacetamide (NMA) using mechanical stirring at room temperature for 15 h. On the other hand, SGO dispersion was made by adding 0.125 g SGO in 10 mL NMA. The PVDF and SGO were blended under consistent mixing for 12 h. On guaranteeing complete homogenous dissolution, the solution was degassed by sonication for 45 min. At last, cast the homogeneous solution into Petri-dishes and evaporate the solvent at 80 °C. The dried polymer films were washed a few times with acetone and demineralized water to expel leftover solvents. Now, the polypyrrole (PPy) was synthesized by oxidative polymerization method on dried PVDF/SGO composite polymer membranes. To carry out the polymerization of pyrrole, the polymer membrane was kept into a beaker containing pyrrole solution under constant stirring. To this pyrrole solution, an equal amount of prepared FeCl_3_ solution was added slowly in 1:1 ratio. After 30 min of stirring, a black layer of polymerized PPy was deposited over the PVDF/SGO composite polymer membranes. The PVDF/SGO/PPy membrane was left in the beaker for digestion. After that, the PVDF/SGO/PPy membrane was washed with demineralized water (DMW) and dried. Finally, to developed the IPMC actuator, the electroding of platinum metal (Pt) on the surface of fabricated PVDF/SGO/PPy membranes was carried as proposed by Inamuddin *et al*. and Ahamed *et al*.^4,7^.

## Results and Discussion

**Figure 1:**
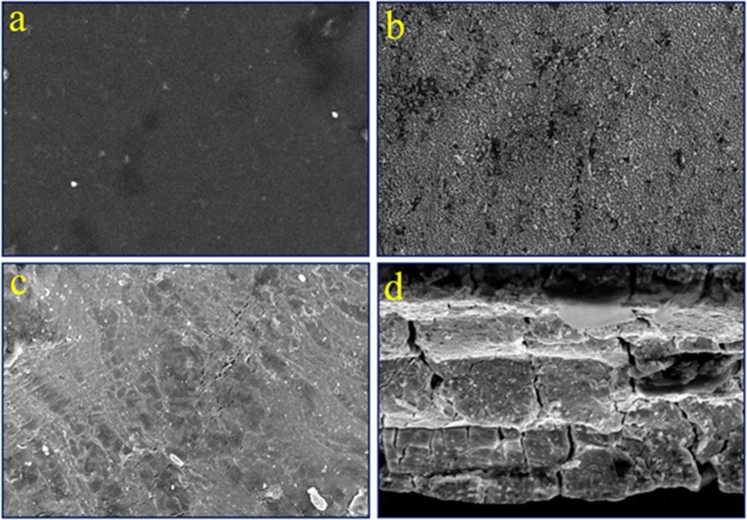
Surface morphology and cross-section view of a fabricated IPMC membrane.

The as-fabricated PVDF/SGO/PPy/Pt layered configuration is revealed in Fig. 1. Figure 1(a) shows the surface of the PVDF/SGO ionomeric composite membrane. The oxidative polymerized deposited PPy on PVDF/SGO composite polymer membrane exhibits a distinctive feature of conducting polymers, showing the squashed structure with granular domains (Fig. 1b). As expected, the Pt electrode layer presents the uniformly smooth surface characteristic of the conventionally electroless plating of Pt ions on the actuator surfaces (Fig. 1c). The cross-sectional view of PVDF/SGO/PPy/Pt structure is shown in Fig. 1(d). As can be seen, the PPy layer of conducting polymer has represented a layered structure over PVDF/SGO composite membrane, while electrode surface of Pt tightly adheres to the PVDF/SGO/PPy composite ionomeric membrane. The strong association among the PPy and SGO benefits in the polymerization of pyrrole monomers on PVDF/SGO membrane. Indeed, the Pt electroding and PPy layers still intimately fasten together after investigation actuation performance, and similar morphology was observed.

**Figure 2:**
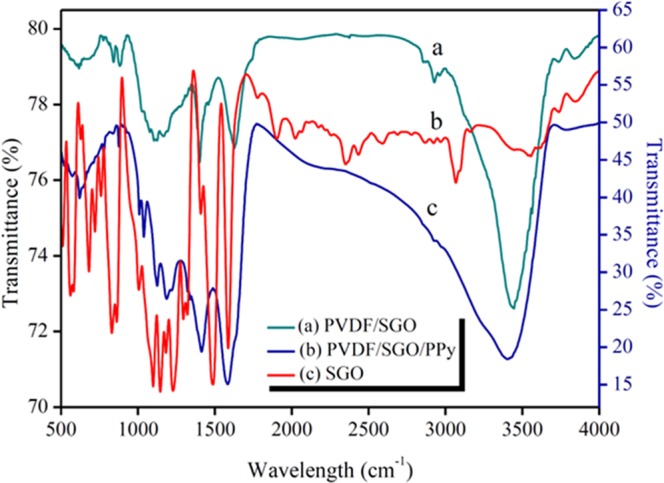
Fourier transform infrared spectroscopic spectra of (**a**) PVDF/SGO, (**b**) PVDF/SGO/PPy and (**c**) SGO.

Figure 2 demonstrates the FTIR spectra of PVDF/SGO, PVDF/SGO/PPy, and SGO. As shown in Fig. 2(a,b), the characteristic peaks at ~1400 and ~854 cm^−1^, which are assigned to the C-F stretching vibration and an absorbance band at ~1180 cm^−1^ was assigned due to the C-C bond of the PVDF^29^. The absorption band at ~3440 cm^−1^ shown in Fig. 2(a,b) is due to the O-H stretching of -SO_3_H and absorbed moisture. The characteristic pinnacles of -SO_3_H show up at ~1088 and ~1036 cm^−1^ demonstrating the presence of O = S = O and S = O stretching, separately, into PVDF/SGO/PPy (Fig. 2b). The peaks around ~1032 and ~1140 cm^−1^ are assigned towards bending vibrations, and at ~1270 cm^−1^ is due to C−N stretching of polypyrrole (Fig. 2b). Another band at ~871 cm^−1^ is due to = C-H in-plane vibration^30^. The peaks appear at ~1500 and ~1460 cm^−1^ are attributed to C=C and C-C stretching vibrations, respectively. The synthesis of SGO from graphite was affirmed by the typical peaks at 3400, 1600, 1220 and 1060 cm^−1^, which are attributed to -OH from intercalated water molecules, C=O and C-OH from a carboxylic acid and C-O stretching, respectively (Fig. 2c)^31–33^. The two significant peaks at ~1113 and ~1017 cm^−1^ are ascribed to the symmetric and asymmetric modes of stretching of O=S=O in -SO_3_H groups and one more little peak at ~815 cm^−1^ is related to the stretching vibration of p-disubstituted phenyl groups (Fig. 2c).

Solvent or water uptake is required for the tip displacement of ionic polymer metal composite membrane actuator due to the migration of cations along with hydrated molecules under the electrical stimulus. Additionally, the high WU of ion exchange polymer membrane also helps to improve the proton conductivity and the dielectric constant of the IPMC membrane^34^. To check the WU, both the IPMCs were dipped in demineralized water at room temperature (R.T.) and 60 °C and WU were measured after an interval of 24 h. The maximum WU for PVDF/SGO/Pt and PVDF/SGO/PPy/Pt ionic polymer metal composite membrane at R.T. after 24 h of immersion was found to be 30 and 40%, respectively, which further increases with the increase in temperature (60 °C) and found to be 34 and 48%, respectively after 24 h (Table 1). The higher WU in case of PVDF/SGO/PPy/Pt film might be because of the presence of PPy, a layer that expands the porosity on the polymer film surface and builds the site capacity for water atoms to be retained. The high IEC is also a characteristic feature of the IPMC, which lead to the Pt particles embedded deep within the pores of the membrane by the electroless plating methods. Also, it can increase the capacitance and the electric current^35^ and reduces the resistance of an ionic polymer metal composite membrane^36^. As shown in Table 1 the IEC for PVDF/SGO and PVDF/SGO/PPy/Pt ionomeric polymer membranes was 0.89 and 1.4 meq g^−1^ of the dry membrane at room temperature. The outcomes got from the examination of WU, and IEC of the PVDF/SGO/PPy/Pt ionic polymer metal composite actuator (Table 1) suggested that the fabricated IPMC membrane have higher water and ionic substance contrasted with Nafion based ionic polymer metal composite actuator^4^. Higher WU and IEC of PVDF/SGO/PPy/Pt IPMC membrane confirm the better execution with respect to other regular polymer based ionic polymer metal composite actuators. High proton conductivity is required to increase the electric current of an IPMC. High capacitance and electric current have been reported to amplify the actuation performance of the IPMC actuator under AC and DC voltages^37^. The PC delivered by the PVDF/SGO/PPy/Pt IPMC membrane (4.251 × 10^−2^ S cm^−1^ at room temperature (R.T.) was higher than that of PVDF/SGO/Pt membrane (3.414 × 10^−3^ S cm^−1^ at R.T.) which may be due to the presence of electrical conductive PPy and -SO_3_H groups in the PVDF/SGO/PPy/Pt ionic polymer metal composite membrane (Table 1). From the PC analysis, it was also investigated that the PVDF/SGO/PPy/Pt ionic polymer metal composite membrane shows the higher value of PC along with the higher value of WU and IEC, which demonstrates the critical role of water molecules and IEC in proton conduction. The IPMC with higher IEC and PC can outcome in a larger amount of water moving in the direction of the cathode by the action of hydrated cations electro-osmosis and electrophoresis drag of water, consequent to larger bending deformation^38^.

**Table 1:** WU, IEC and PC of PVDF/SGO/Pt and PVDF/SGO/PPy/Pt ionic polymer metal composite membranes.

Membranes	WU% at R.T (24 h)	WU% at 60 °C (24 h)	IEC (meq g^−1^ of dry ion exchanger)	PC (S cm^−1^)
PVDF/SGO/Pt	30	34	0.89	3.414 × 10^−3^
PVDF/SGO/PPy/Pt	40	48	1.4	4.251 × 10^−2^

**Figure 3:**
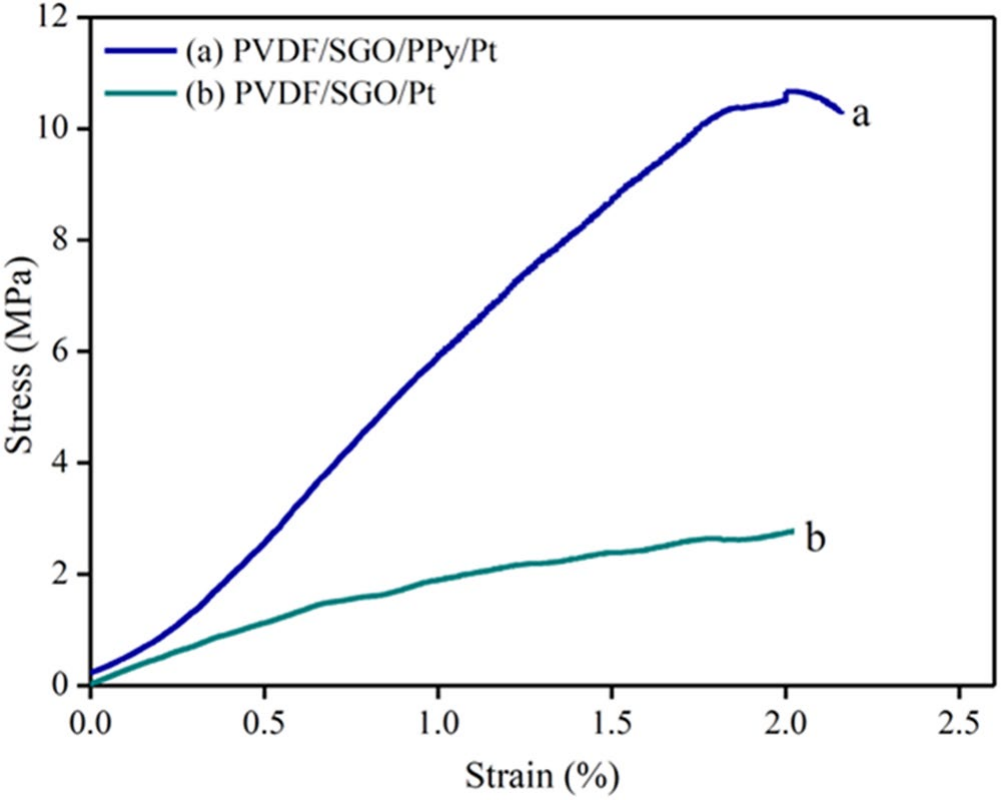
The tensile strength of pure (**a**) PVDF/SGO/PPy/Pt and (**b**) PVDF/SGO/Pt ionic polymer metal composite membrane actuators.

The stress-strain curve was obtained to demonstrate the mechanical strength of PVDF/SGO/Pt and PVDF/SGO/PPy/Pt membranes, as shown in Fig. 3. Both the film of sizes 0.15 mm thick and 20 mm width were settled into the universal testing machine with settled measure length of 25 mm. As the films were prolonged with loads, the uprooting of two ends points was consistently recorded, accomplish the strain. Figure 3 demonstrates the stress-strain curves of the PVDF/SGO/Pt and PVDF/SGO/PPy/Pt-based IPMC, and Table 2 shows their mechanical properties. The tensile strength and young modulus of PVDF/SGO/Pt membrane were 10.8 and 178 MPa, respectively, whereas 28.5 and 520 MPa, respectively, for PVDF/SGO/PPy/Pt membrane. The stress-strain analysis demonstrate that the increased mechanical strength of the proposed PVDF/SGO/PPy/Pt ionic polymer metal composite membrane actuator was due the deposition of PPy polymer layer, which increases the mechanical stability due strong chemical interaction and sufficient amount of π-electrons in the SGO which promotes π−π stacking interactions with PPy within the proposed material composition.

**Table 2:** Mechanical properties of PVDF/SGO/PPy/Pt and PVDF/SGO/Pt membrane actuators.

Membranes	Young’s modulus (MPa)	Tensile strength (MPa)	Elongation at break (%)
PVDF/SGO/Pt	178 ± 0.45	10.8	2.02
PVDF/SGO/PPy/Pt	520 ± 0.41	28.5	2.4

**Figure 4:**
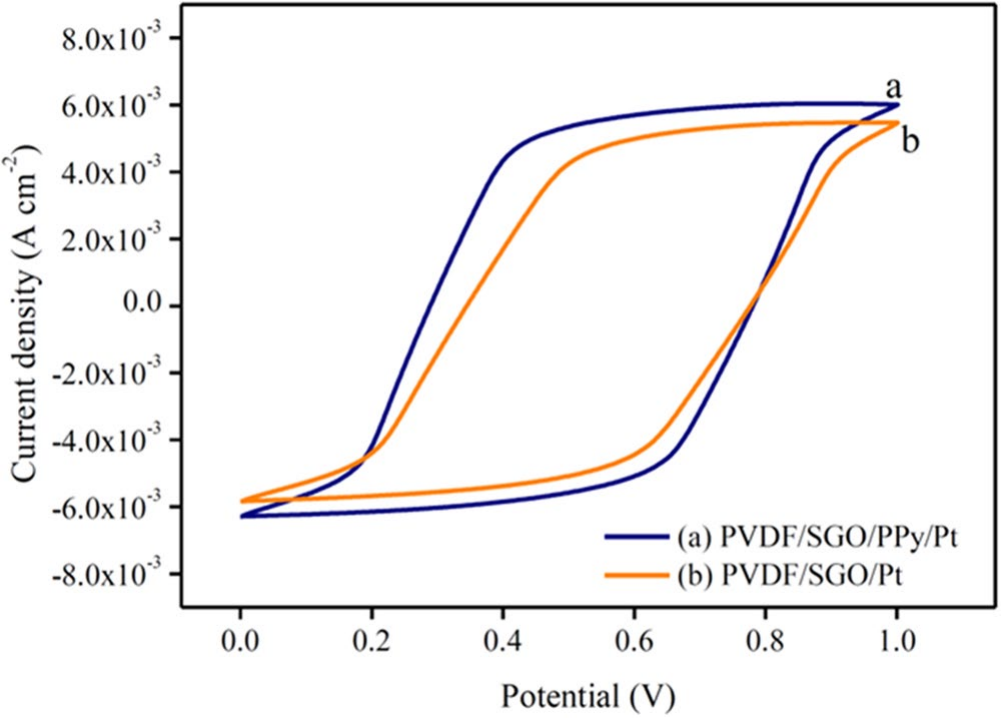
The current-voltage behaviour of (**a**) PVDF/SGO/PPy/Pt and (**b**) PVDF/SGO/Pt ionic polymer metal composite membrane actuator.

The electrochemical characteristics of PVDF/SGO/Pt and PVDF/SGO/PPy/Pt ionic polymer metal composite membrane actuators were investigated by cyclic voltammetric curve (CV) as given in Fig. 4. The symmetric shape of the I-V hysteresis curves for the fabricated IPMC can be assigned to admirable charge allocation, which increases the rate of ionic transfer in PVDF/SGO/PPy/Pt membrane requisite for the enhanced performance of ionic polymer composite actuator. The CV curve shows that the PVDF/SGO/PPy/Pt membrane has a higher value of current density because SGO and PPy increase the sulfuric acid groups and surface area and conductivity in the proposed composition. This further increase the uniformity of the Pt electrode layer deposited on the membrane surface with the increase in the PC and IEC. With the deposition of the PPy layer within the PVDF/SGO/PPy/Pt ionic polymer metal composite membrane, the shape of the CV curve became more rectangular compared with PVDF/SGO/Pt ionic polymer metal composite membrane actuator (Fig. 4b). This rectangular-shaped CV curve may be related with the electrochemical double-layer capacitance mechanism of the SGO and PPy. Furthermore, PVDF/SGO/Pt IPMC membrane exhibited the large CV area, which implies that a sufficient amount of π-electrons in the SGO promotes π−π stacking interactions with PPy within the proposed material composition, resulting in enhanced charge transfer^39^. The electrical investigations uncover that the watched current density of the created economical ionic polymer metal composite film is amazingly higher than a few other reported so far ionic polymer metal composite^5,6^. The enhanced electrochemical properties in the projected ionic polymer metal composite membrane actuator may be due to the addition of electrically conductive PPy layer via polymerization of pyrrole monomer on the ionomeric membrane surface. The current density also reflects the energy storage aptitude of fabricated materials which is obligated for the actuation execution by bigger disfigurement of ionic polymer metal composite actuator. These outcomes likewise uncover that the thick Pt cathodes layer with a very nano dispersed structure in SPEES/PVDF/SGO/Pt film actuator may offer high current density with a vast interfacial zone because of SGO, which is required to be favourable in enhancing the actuation execution of manufactured ionic polymer metal composite actuator.

**Figure 5:**
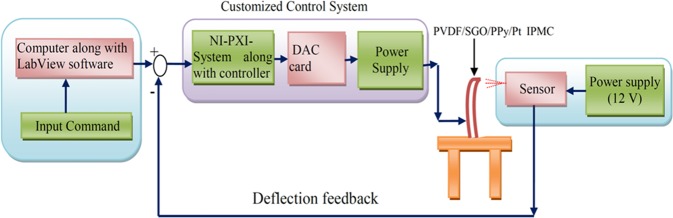
Schematic testing setup to measure the tip displacement of PVDF/SGO/Pt and PVDF/SGO/PPy/Pt ionic polymer metal composite membrane actuator.

The schematic illustration of an experimental test setup to demonstrate the tip displacement of the fabricated IPMC is shown in Fig. 5. The preferred controlled voltage (±5 V) was sent to the IPMCs with the help of this setup. A code was designed with Lab view software for controlling the voltage, integrated with proportional integral derivative (PID) control system, and an algorithm was also developed to achieve the desired deflection. The tip displacement under applied voltage was monitored by using a laser displacement sensor. The data conversion can take place by converting the data from RS-485 to RS-232 communication protocol interfaced with NI-PXI system and NI visa interfaces software module in a Lab-view VI to attain the proper announcement between input command and sensor.

**Figure 6:**
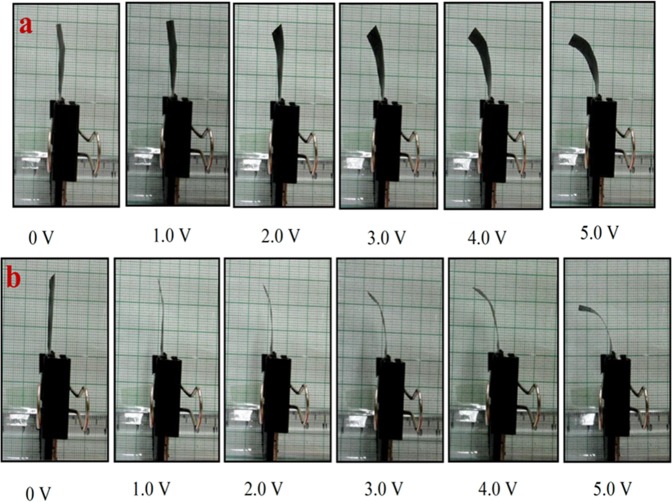
Successive deflection behaviour of (**a**) PVDF/SGO/Pt and (**b**) PVDF/SGO/PPy/ ionic polymer metal composite membrane actuators.

**Figure 7:**
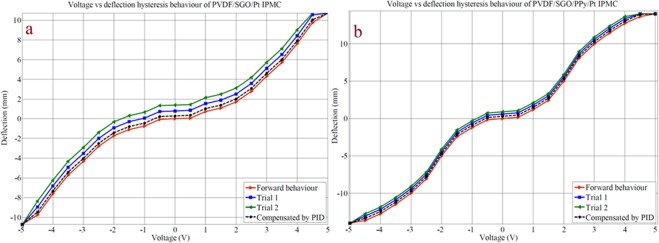
Voltage versus deflection hysteresis behaviour of (**a**) PVDF/SGO/Pt and (**b**) PVDF/SGO/PPy/Pt ionic polymer metal composite membrane actuator.

The stepwise tip displacement of PVDF/SGO/PPy/Pt and PVDF/SGO/Pt ionic polymer metal composite membrane actuators under voltages (0–5 V DC) is shown in Fig. 6. This successive deflection experiment was repeated for 3 × 10 (30) times at the same applied potential of 0–5 V DC for both the IPMCs to attain the performance after multiple repeats. The experimental deflection data (for 10 repeats) of both the IPMC membranes are given in Tables 3 and 4, respectively, while the deflection data for the remaining trials are provided in Table S1–S4. To analyze the deflection hysteresis behaviours the average value of each 10 experiments were taken as one trial at multiple repeats and plotted as shown in Fig. 7(a,b). The results observed from the hysteresis analysis show that both the IPMC membranes show steady-state behaviour with the increase in applied voltage form 0–5 but when the voltage was lowered, the membranes provide some deflection error (hysteresis), *i*.*e*. did not follow the same path to returns to its initial position. To minimize this deflection error PID control system tuned in Labview software was used, which reduced the hysteresis up to 80%. Moreover, the hysteresis results also reveal that (Fig. 7(a,b)) with the repetition of the tip displacement experiment the deflection error was increases and hysteresis curve became more broaden for PVDF/SGO/Pt IPMC membrane, which reveals the lower performance of the IPMC after multiple repeats. While for PVDF/SGO/PPy/Pt there is no significant effect on the hysteresis curve, however, the repetition of deflection may be due to the additional PPy polymer layer, which increases the charge transfer due to increase in surface area for a chemical reaction.

**Table 3:** Experimental tip displacement data of PVDF/SGO/PPy/Pt ionic polymer metal composite membrane actuator.

Deflection (mm)	Voltage (V)										
	0 V	0.5 V	1.0 V	1.5 V	2.0 V	2.5 V	3.0 V	3.5 V	4.0 V	4.5 V	5.0 V
d1	0	0.12	1.20	2.51	4.58	7.95	9.98	11.51	12.54	13.51	14.00
d2	0	0.18	1.02	2.57	4.67	7.84	10.57	10.85	13.12	13.65	14.01
d3	0	0.15	1.65	2.26	4.87	7.65	9.98	11.32	12.68	13.66	14.10
d4	0	0.14	1.35	2.74	4.68	8.24	10.24	11.45	12.85	13.41	13.90
d5	0	0.16	0.98	2.65	5.24	8.34	9.87	11.52	12.56	13.54	13.96
d6	0	0.17	1.00	1.98	5.26	8.64	9.67	11.34	12.85	13.62	14.00
d7	0	0.18	1.24	2.21	4.87	8.24	9.64	11.25	12.67	13.33	13.94
d8	0	0.17	1.02	2.34	4.98	7.68	9.57	12.00	12.57	13.80	13.95
d9	0	0.14	1.34	2.49	5.35	8.24	10.54	11.86	12.67	13.88	14.01
d10	0	0.13	1.32	2.68	5.34	7.65	9.34	11.35	12.65	13.75	13.99

**Table 4:** Experimental tip displacement data of PVDF/SGO/Pt ionic polymer metal composite membrane actuator.

Deflection (mm)	Voltage (V)										
	0 V	0.5 V	1.0 V	1.5 V	2.0 V	2.5 V	3.0 V	3.5 V	4.0 V	4.5 V	5.0 V
d1	0	0.08	0.91	1.01	1.85	2.85	4.65	5.56	7.58	9.54	10.00
d2	0	0.05	0.75	0.99	2.02	2.68	4.35	6.21	7.56	9.65	10.21
d3	0	0.07	0.84	1.11	1.67	2.84	4.24	5.94	7.65	9.86	11.10
d4	0	0.04	0.64	1.02	1.67	2.64	3.95	5.46	7.35	9.84	10.54
d5	0	0.05	0.82	1.00	1.94	2.84	3.87	5.32	7.64	9.64	10.64
d6	0	0.02	0.52	0.95	1.43	3.01	4.08	6.04	7.98	9.65	10.85
d7	0	0.03	0.81	1.21	1.74	2.73	4.62	6.21	7.24	9.86	10.90
d8	0	0.07	0.41	1.05	1.64	2.74	4.61	5.31	7.91	9.75	10.57
d9	0	0.06	0.67	1.21	1.37	2.64	3.98	5.02	7.61	10.21	11.23
d10	0	0.05	1.00	1.41	1.87	3.05	4.82	6.05	7.84	9.64	11.14

**Figure 8:**
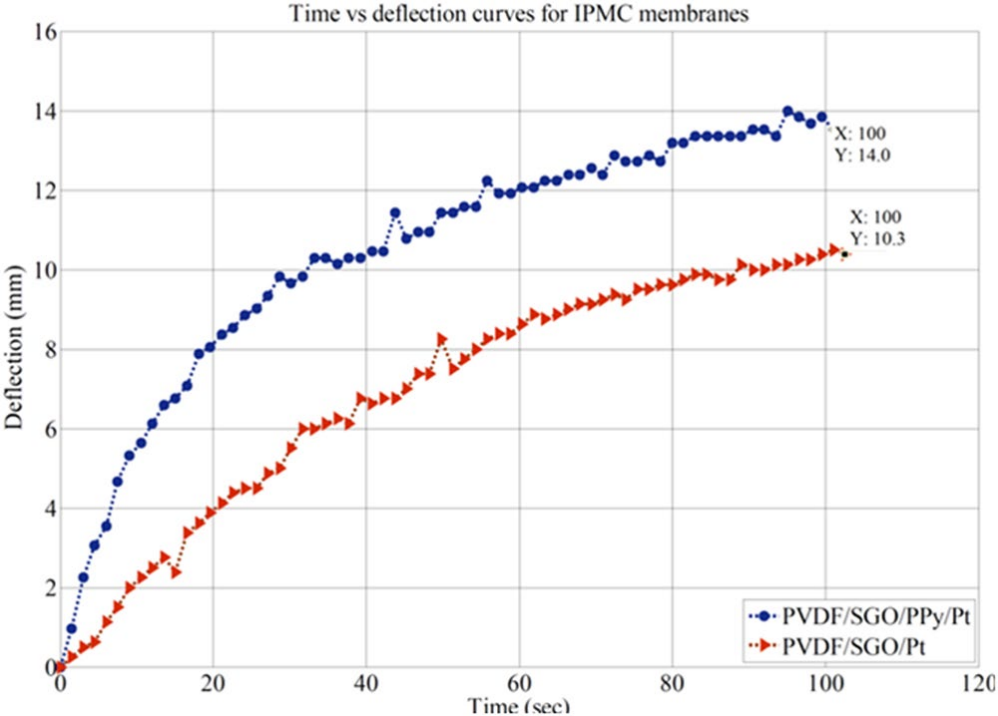
Bending response over time for both the fabricated IPMC membranes.

The result of the bending response of PVDF/SG/Pt and PVDF/SGO/PPy/Pt ionic polymer metal composite membrane actuator over time at 5 V is shown in Fig. 8. As shown in Fig. 8, the tip displacement for both PVDF/SG/Pt and PVDF/SGO/PPy/Pt IPMC membranes increases with time and reaches to maximum displacement value up-to 10 and 14 mm, respectively, after that the deflection was saturated. From the bending response of the IPMC, it was clear that the tip displacement was increasing continuously for both the membranes up to 100 s at a constant voltage of 5 V DC. Here for the developed IPMC, the maximum allowed limit of voltage was found to be 5 V DC. Further increase of applied voltage gets the IPMC dried and  burns after 5 V DC, so that bending response was recorded at the most possible higher potential difference between electrodes (voltage) as 5 V DC. The obtained results confirm that precise bending behaviour and large tip displacement of PVDF/SGO/PPy/Pt IPMC actuator was much better than PVDF/SGO/Pt IPMC just because of the presence of conductive polymer layer of PPy.

**Figure 9:**
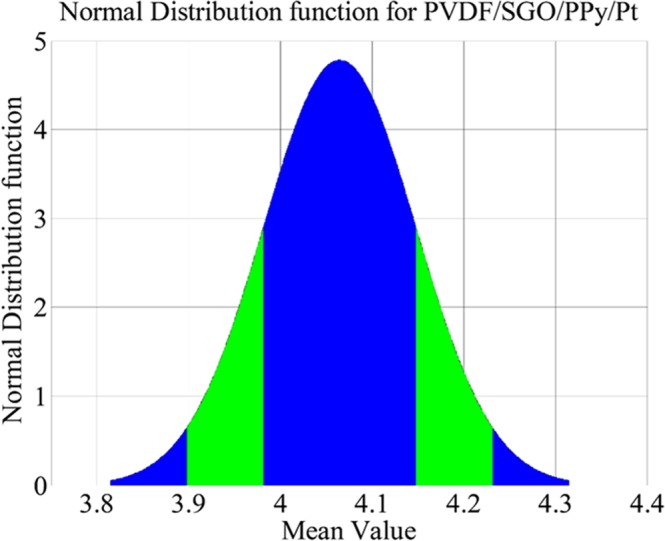
The normal distribution function for PVDF/SGO/PPy/Pt ionic polymer metal composite membrane actuator.

To demonstrate the generated force behaviour of PVDF/SGO/ Pt and PVDF/SGO/PPy/Pt ionic polymer metal composite membrane actuators a digital load cell was taken, where the tip of ionic polymer composite membrane contacts the pan of load cell upon electrical stimulus through NI-PXI system. The PVDF/SGO/Pt and PVDF/SGO/PPy/Pt ionic polymer metal composite membrane actuator bends to generate the maximum load carrying capability up-to 0.30 and 0.41 g at 5 V DC, respectively. For finding the performance after repetition of these IPMC membranes, the several trials of load characterizations were conducted and the force data were noted as provided in Tables 5 and 6. The force data at different voltages shows that for all the repeated experiments PVDF/SGO/PPy/Pt ionic polymer metal composite membrane actuator provided large force than the PVDF/SGO/Pt ionic polymer metal composite membrane actuator. After taking an average of ten force values, the mean force value for both the IPMCs was obtained to calculate the standard deviation (SD). The SD for PVDF/SGO/PPy/Pt and PVDF/SGO/Pt ionic polymer metal composite membrane actuators was found to be 0.734 and 0.525, respectively. The normal distribution for the PVDF/SGO/PPy/Pt ionic polymer metal composite membrane actuator was plotted, as shown in Fig. 9. The normal distribution curve suggested that the narrow shape confirm the PVDF/SGO/PPy/Pt IPMC has less error and a good repeatability of the forced behaviour at 5 V DC.

**Table 5:** Experimental generated force data for PVDF/SGO/PPy/Pt IPMC membrane.

Force (mN)	Voltage (V)					
	0 V	1 V	2 V	3 V	4 V	5 V
F1	0	0.1078	1.1858	1.94824	3.12816	4.1111
F2	0	0.1176	0.96922	2.26772	3.18892	4.06308
F3	0	0.0833	1.0633	2.09916	3.3565	4.15618
F4	0	0.13328	1.29458	1.9747	2.9792	3.94352
F5	0	0.09604	0.9457	2.29418	2.9841	4.04348
F6	0	0.10094	1.00352	2.4353	3.675	4.11992
F7	0	0.10976	1.31418	1.8571	2.92432	4.05328
F8	0	0.09996	1.21618	2.00606	2.98802	4.1013
F9	0	0.10094	1.01332	2.12072	2.96156	3.90628
F10	0	0.09702	1.7101	2.36474	2.9498	4.1503
Operating voltage						5.0 V
Mean						4.06484 mN
Standard deviation						0.525
Repeatability						98.72%

**Table 6:** Experimental generated force data for PVDF/SGO/Pt IPMC membrane.

Force (mN)	Voltage (V)					
	0 V	1 V	2 V	3 V	4 V	5 V
F1	0	0.01323	0.4263	1.38376	1.97176	2.94490
F2	0	0.030772	0.34986	1.42884	1.99332	2.95176
F3	0	0.02352	0.56252	1.82672	1.99038	2.77732
F4	0	0.06272	0.35672	1.29752	1.9453	2.36180
F5	0	0.06076	0.45276	1.39552	1.8277	2.70088
F6	0	0.07448	0.85652	1.3475	2.07858	2.72832
F7	0	0.05978	0.35672	1.35926	1.85808	2.31672
F8	0	0.04116	0.53508	1.4455	1.97176	2.33730
F9	0	0.04312	0.34692	1.35926	1.96098	2.69010
F10	0	0.04018	0.35378	1.65326	2.08152	2.87532
Operating voltage						5.0 V
Mean						2.6684 mN
Standard deviation						0.734
Repeatability						90.51%

**Figure 10:**
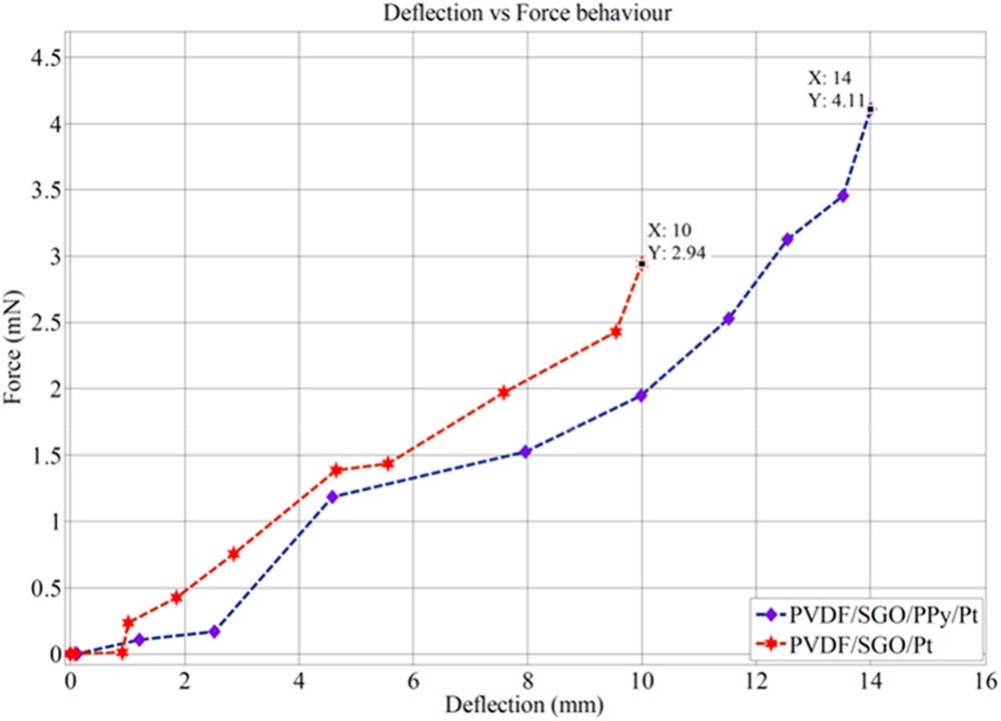
Deflection versus force behaviour for both the fabricated IPMCs.

**Figure 11:**
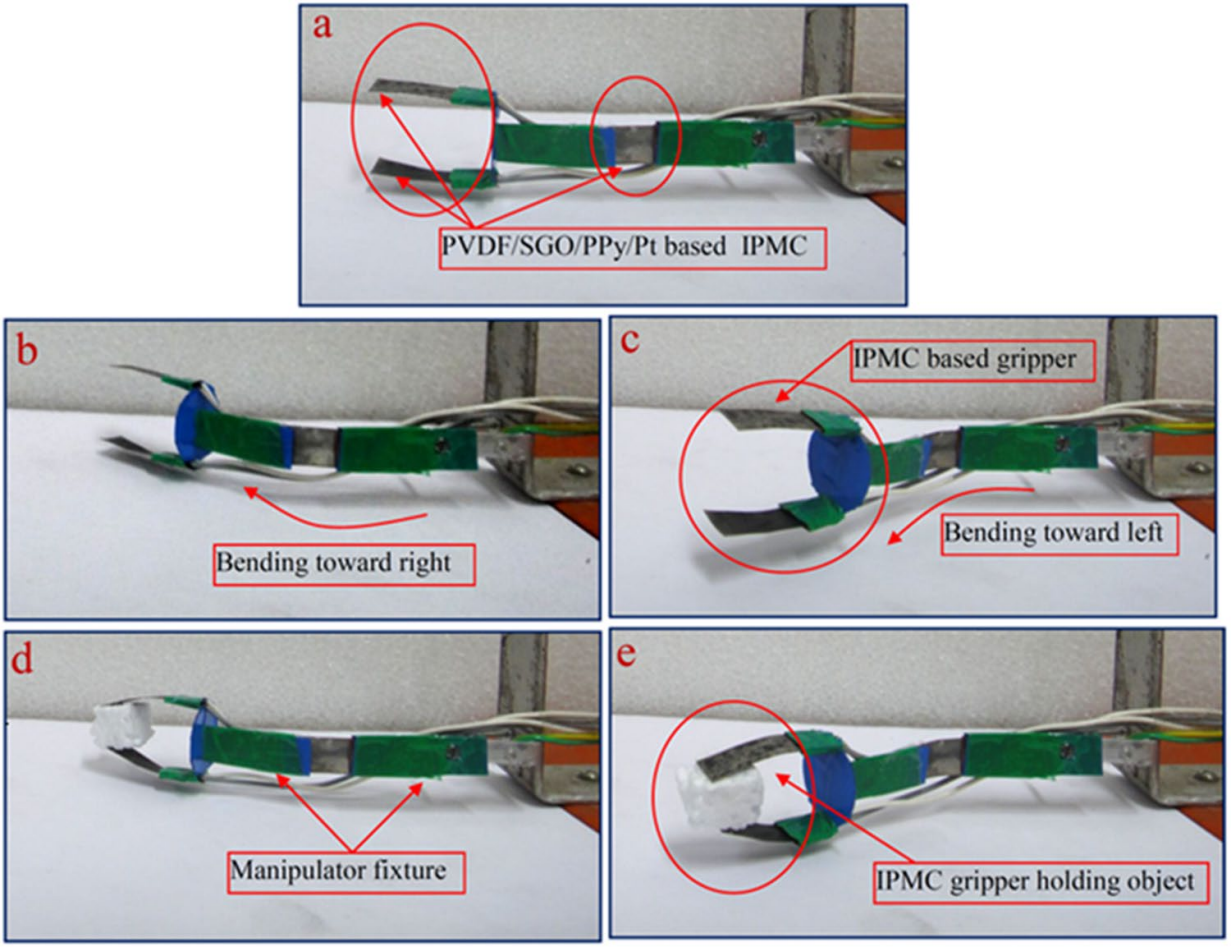
Flexible link manipulator based on PVDF/SGO/PPy/Pt ionic polymer metal composite membrane actuator.

Furthermore, Fig. 10 shows that the deflection versus generated force of the PVDF/SGO/PPy/Pt and PVDF/SGO/Pt ionic polymer metal composite membrane actuators are also proportional to the applied electric voltage (0–5 V) because the generated force was indeed proportional to the electric field like the tip displacement (Tables 5 and 6). Therefore, the large tip displacement reflects the larger generated force. Thus, due to large tip displacement, the PVDF/SGO/PPy/Pt ionic polymer metal composite membrane actuator generated higher force than PVDF/SGO/Pt ionic polymer metal composite membrane actuator. After characterization, a novel flexible link manipulator using PVDF/SGO/PPy/Pt-based IPMC membrane was developed as shown in Fig. 11. In the developed manipulator a PVDF/SGO/PPy/Pt ionic polymer metal composite membrane actuator was considered to be as flexible joint and two other IPMC finger based microgripper was also integrated at the end of flexible links. The flexible IPMC joint provides the bi-directional bending of the link manipulator for manipulating the object from one position to another position. The PVDF/SGO/PPy/Pt IPMC membrane based microgripper holds the object by providing the applied voltage (5 V DC). This shows the potential of handling small objects.

## Conclusions

This paper presented the novel composition for the development of a new ionic polymer metal composite membrane actuator based on PVDF and SGO blend that was coated with PPy/Pt electrode. The PVDF/SGO/PPy/Pt membrane inveterate high PC, IEC and good WU, high current density and higher tensile modulus than that of PVDF/SGO/Pt ionic polymer metal composite membrane actuator. Beyond the improved characteristic parameters, normal distribution curve reveals the excellent repeatability of PVDF/SGO/PPy/Pt ionic polymer metal composite membrane actuator. The composition of PVDF/SGO/PPy/Pt ionic polymer metal composite membrane actuator presented in this paper may pave the way to amend and enhance the properties of IPMC with a straightforward blending process, which can offer more cost-effective materials for the development of IPMCs that can be used in dexterous handling devices in robotic manipulation.

## Supplementary information

Supplementary Tables
