# Supplementary material for: Polyvinylidene fluoride/sulfonated graphene oxide blend membrane coated with polypyrrole/platinum electrode for ionic polymer metal composite actuator applications
Source: Sci Rep. 2019 Jul 8;9:9877. doi: 10.1038/s41598-019-46305-6 (PMC6614476; doi:10.1038/s41598-019-46305-6)
Supplement: Supplementary file 1 — Supplementary Tables [file 41598_2019_46305_MOESM1_ESM.docx]

**Polyvinylidene fluoride/sulfonated graphene oxide blend membrane coated with polypyrrole/platinum electrode layers for ionic polymer metal composite actuator applications**

Inamuddin and Heba Abbas Kashmery

Chemistry Department, Faculty of Science, King Abdul Aziz University, Jeddah 21589, Saudi Arabia

Corresponding author E-mail: [inamuddin@rediffmail.com](mailto:inamuddin@rediffmail.com) (Inamuddin)

**Table S1.** Experimental tip displacement data of PVDF/SGO/Pt IPMC actuator for trial 2.

| Deflection (mm) | Voltage (V) | | | | | | | | | | |
| --- | --- | --- | --- | --- | --- | --- | --- | --- | --- | --- | --- |
|  | **0 V** | **0.5V** | **1.0V** | **1.5V** | **2.0V** | **2.5V** | **3.0V** | **3.5V** | **4.0V** | **4.5 V** | **5.0 V** |
| d1 | 0.2599 | 0.5772 | 1.1012 | 1.5542 | 3.9158 | 5.4777 | 8.4046 | 6.3735 | 10.9326 | 9.5979 | 10.1156 |
| d2 | 0.2201 | 0.0709 | 0.966 | 4.1467 | 3.326 | 3.8412 | 5.3721 | 8.7544 | 11.2445 | 9.7271 | 10.265 |
| d3 | 0.0861 | 0.4459 | 0.9225 | 1.4797 | 4.318 | 5.8592 | 6.3734 | 9.1346 | 9.6436 | 9.9419 | 11.1302 |
| d4 | 0.6521 | 0.0628 | 3.3669 | 1.9718 | 2.1404 | 4.8733 | 7.7704 | 7.4675 | 8.4608 | 9.9006 | 10.5401 |
| d5 | 0.0965 | 0.639 | 3.2154 | 1.9749 | 2.5315 | 4.5518 | 4.9414 | 7.9242 | 10.251 | 9.775 | 10.6952 |
| d6 | 0.2365 | 0.2621 | 0.9763 | 1.3694 | 1.5091 | 4.0792 | 5.0807 | 9.225 | 11.6505 | 9.6679 | 10.9572 |
| d7 | 0.6391 | 0.6329 | 3.9961 | 4.6446 | 5.5985 | 5.746 | 8.332 | 7.1438 | 9.2801 | 9.9382 | 11.0695 |
| d8 | 0.3558 | 0.2452 | 2.8823 | 3.8438 | 5.5229 | 6.3348 | 4.8844 | 7.7142 | 11.8082 | 9.8074 | 10.5782 |
| d9 | 0.4381 | 0.4741 | 0.951 | 4.146 | 1.8656 | 5.5548 | 5.178 | 5.47 | 8.3994 | 10.2696 | 11.4061 |
| d10 | 0.4152 | 0.7177 | 1.2772 | 4.0131 | 3.7403 | 4.6779 | 7.1872 | 8.1138 | 8.2849 | 9.6605 | 11.1655 |

**Table S2.** Experimental tip displacement data of PVDF/SGO/Pt IPMC actuator for trial 3.

| Deflection (mm) | Voltage (V) | | | | | | | | | | |
| --- | --- | --- | --- | --- | --- | --- | --- | --- | --- | --- | --- |
|  | **0 V** | **0.5V** | **1.0V** | **1.5V** | **2.0V** | **2.5V** | **3.0V** | **3.5V** | **4.0V** | **4.5 V** | **5.0 V** |
| d1 | 0.3021 | 0.6708 | 3.3695 | 1.7259 | 3.6352 | 6.3838 | 6.9154 | 8.4092 | 10.5865 | 9.5408 | 10.1274 |
| d2 | 0.0255 | 0.6499 | 3.8341 | 1.5153 | 2.656 | 3.0173 | 8.0073 | 7.8417 | 7.8457 | 9.7572 | 10.3154 |
| d3 | 0.215 | 0.2091 | 4.309 | 4.6785 | 2.9923 | 4.7127 | 5.9414 | 6.6775 | 11.0865 | 9.9663 | 11.2233 |
| d4 | 0.5885 | 0.1308 | 2.1928 | 1.1988 | 2.9437 | 2.7632 | 5.4024 | 8.9327 | 10.1074 | 9.8985 | 10.5496 |
| d5 | 0.2544 | 0.6529 | 1.1633 | 3.7851 | 5.5678 | 5.8947 | 5.8532 | 7.6893 | 8.1942 | 9.6479 | 10.6655 |
| d6 | 0.0612 | 0.4884 | 3.4963 | 3.9259 | 2.4318 | 5.8491 | 5.1181 | 7.5551 | 11.4614 | 9.7314 | 10.9524 |
| d7 | 0.3753 | 0.0418 | 2.1564 | 2.983 | 3.0000 | 3.600 | 8.3884 | 7.1091 | 8.0505 | 9.8842 | 10.9968 |
| d8 | 0.2694 | 0.7279 | 3.8157 | 2.5905 | 3.2982 | 5.4975 | 6.503 | 6.1982 | 10.3732 | 9.8513 | 10.678 |
| d9 | 0.543 | 0.4384 | 2.1776 | 5.1832 | 4.2415 | 4.9002 | 5.0102 | 7.138 | 9.8124 | 10.2843 | 11.2998 |
| d10 | 0.3853 | 0.4498 | 4.359 | 3.0282 | 2.4525 | 6.5001 | 6.5689 | 7.8078 | 8.4983 | 9.6752 | 11.1991 |

**Table S3.** Experimental tip displacement data of PVDF/SGO/PPy/Pt IPMC actuator for trial 2.

| Deflection (mm) | Voltage (V) | | | | | | | | | | |
| --- | --- | --- | --- | --- | --- | --- | --- | --- | --- | --- | --- |
|  | **0 V** | **0.5V** | **1.0V** | **1.5V** | **2.0V** | **2.5V** | **3.0V** | **3.5V** | **4.0V** | **4.5 V** | **5.0 V** |
| d1 | 0.0231 | 0.7535 | 1.2862 | 3.7452 | 7.0323 | 10.4364 | 10.4408 | 13.5839 | 13.9878 | 13.6346 | 14.006 |
| d2 | 0.2348 | 0.5213 | 4.4452 | 6.4969 | 6.7656 | 9.0607 | 14.2744 | 10.9626 | 13.7041 | 13.7651 | 14.0877 |
| d3 | 0.5297 | 0.4632 | 2.819 | 5.8958 | 4.9005 | 10.8888 | 11.9345 | 15.3389 | 16.1317 | 13.6908 | 14.1024 |
| d4 | 0.4558 | 0.3605 | 2.3659 | 3.5111 | 7.4756 | 11.4687 | 13.6966 | 13.4828 | 14.2211 | 13.5049 | 13.9816 |
| d5 | 0.1197 | 0.1991 | 2.9618 | 2.6573 | 9.0789 | 11.5096 | 13.1566 | 12.8672 | 13.6767 | 13.7054 | 14.1239 |
| d6 | 0.6736 | 0.7032 | 3.9584 | 4.8683 | 8.8048 | 10.0647 | 10.4279 | 12.0456 | 12.8744 | 13.7227 | 14.0429 |
| d7 | 0.3843 | 0.2733 | 2.0622 | 5.7312 | 5.3297 | 8.4603 | 10.6431 | 13.7888 | 15.9241 | 13.4693 | 14.0066 |
| d8 | 0.1778 | 0.4218 | 1.8976 | 2.8201 | 6.4188 | 10.5559 | 9.7899 | 14.3339 | 14.5885 | 13.9697 | 14.0243 |
| d9 | 0.4093 | 0.42 | 5.3019 | 2.6484 | 6.3318 | 12.2692 | 13.0111 | 14.9074 | 14.8525 | 14.0327 | 14.1532 |
| d10 | 0.6476 | 0.7564 | 3.7273 | 5.1074 | 7.6138 | 8.3093 | 12.494 | 11.973 | 16.1841 | 13.7869 | 14.1321 |

**Table S4.** Experimental tip displacement data of PVDF/SGO/PPy/Pt IPMC actuator for trial 3.

| Deflection (mm) | Voltage (V) | | | | | | | | | | |
| --- | --- | --- | --- | --- | --- | --- | --- | --- | --- | --- | --- |
|  | **0 V** | **0.5V** | **1.0V** | **1.5V** | **2.0V** | **2.5V** | **3.0V** | **3.5V** | **4.0V** | **4.5 V** | **5.0 V** |
| d1 | 0.3184 | 0.6935 | 1.9973 | 5.3234 | 6.0815 | 8.5834 | 10.9388 | 15.4608 | 12.9354 | 13.6088 | 14.0786 |
| d2 | 0.1534 | 0.2691 | 2.2142 | 4.5987 | 7.7726 | 8.9868 | 12.6552 | 13.9324 | 15.3098 | 13.7412 | 14.097 |
| d3 | 0.2395 | 0.6133 | 3.6378 | 3.7864 | 5.5318 | 10.4009 | 10.8571 | 14.6411 | 14.2932 | 13.7131 | 14.1965 |
| d4 | 0.0821 | 0.3968 | 4.9126 | 2.9999 | 8.332 | 8.8051 | 13.6124 | 14.2767 | 13.2785 | 13.4926 | 13.9342 |
| d5 | 0.2779 | 0.4079 | 2.9874 | 4.0855 | 6.5179 | 10.3901 | 12.524 | 13.9034 | 15.4591 | 13.5895 | 14.0442 |
| d6 | 0.2444 | 0.5956 | 2.1089 | 2.9178 | 6.5802 | 11.5278 | 12.9377 | 14.3541 | 15.3068 | 13.7067 | 14.0563 |
| d7 | 0.2534 | 0.8199 | 3.3776 | 3.0247 | 5.6876 | 11.9585 | 12.8182 | 13.2386 | 15.8045 | 13.3638 | 13.9885 |
| d8 | 0.6052 | 0.3171 | 3.3189 | 5.5979 | 8.0515 | 10.6109 | 11.448 | 15.4635 | 14.8384 | 13.9022 | 14.0359 |
| d9 | 0.3938 | 0.6381 | 2.9926 | 4.0651 | 5.6301 | 11.2419 | 11.7792 | 12.1323 | 15.918 | 13.9091 | 14.1096 |
| d10 | 0.386 | 0.798 | 1.3791 | 2.8945 | 9.1434 | 9.2807 | 12.0927 | 15.2275 | 14.9591 | 13.7576 | 13.998 |
